# Improving the Diagnosis of Clinically Significant Prostate Cancer with Magnetic Resonance Imaging

**DOI:** 10.5334/jbsr.1438

**Published:** 2018-02-07

**Authors:** Pieter De Visschere

**Affiliations:** 1 Ghent University Hospital, BE

**Keywords:** Prostate, Magnetic Resonance Imaging, Diffusion Weighted Imaging, Dynamic Contrast Enhanced Imaging, Cancer

## Introduction

Magnetic resonance imaging (MRI) is being used increasingly in the diagnosis of prostate cancer (PC). Over the past years, the prostate MRI scan protocol that is recommended by the international guidelines has been changing and also the clinical indications for performing a prostate MRI have evolved. In this paper, the currently considered optimal prostate MRI scan protocol is described, and the accuracy of prostate MRI in the diagnosis of clinically significant PC in patients with elevated prostate specific antigen (PSA) is reviewed.

## Prostate MRI Scan Protocol

Morphological T2-Weighted Imaging (T2-WI) is the basis of any prostate MRI exam. Large and high-grade PC in the prostate can be identified as ill-defined low signal intensity (SI) lesions, contrasting well with the high background SI of the peripheral zone (PZ), but are often more difficult to distinguish in the transition zone (TZ) due to the lower and more heterogeneous background SI of the latter caused by sparser glandular tissue and the more prominent fibromuscular tissue [1,2]. The high and very high SI of what is commonly denoted as *normal* prostate tissue basically represents cystic atrophy and large-gland simple atrophy [3,4,5] whereas pure normal prostate glands are actually iso-intense on T2-WI. This explains why the PZ is iso-intense on T2-WI in young patients [6,7,8].

T2-WI alone is however not sufficient to detect smaller and less aggressive PC and to improve the diagnostic accuracy of prostate MRI, it is necessary to add T1-WI (to detect post-biopsy haemorrhage) and functional imaging techniques. In 2011 the ESUR (European Society of Urogenital Radiology) published guidelines on prostate MRI [3], describing the minimal requirements for a high quality prostate MRI. A multiparametric MRI (mpMRI) approach was promoted, defined as a scan protocol combining morphological T2-WI with at least two functional imaging techniques, such as dynamic contrast-enhanced MRI (DCE-MRI), diffusion-weighted imaging (DWI) and/or MR spectroscopy (MRSI). A mpMRI provided an abundance of information about anatomy, tissue density, neovascularity, vascular permeability, metabolite concentrations, mobility of water molecules, etc … and it was thought that the more information one achieved, the better differentiation would be possible between PC and non-cancerous tissue. Some histological conditions indeed have unique features on mpMRI and are consequently easy to recognize. Cystic atrophy is such a condition: it shows very high SI on T2-WI surrounded by a thin hypo-intense line, bulging on the surrounding tissues, absent contrast enhancement on DCE, high ADC value on DWI and high citrate concentrations on MRSI. Poorly differentiated PC also has typical features: very low SI on T2-WI, (very) low ADC value on DWI, strong contrast enhancement on DCE and high choline concentrations on MRSI [5]. The interpretation of mpMRI may however be difficult because non-cancerous conditions such as adenosis, post-atrophic hyperplasia (PAH) and inflammation may mimic well-differentiated PC. These conditions all show ill-defined iso- to hypointense SI on T2-WI, moderate ADC values on DWI, moderate to strong contrast enhancement on DCE and moderate citrate and choline concentrations on MRSI. Despite the abundance of information provided by the different morphological and functional imaging sequences, a mpMRI protocol may thus not avoid that benign mimickers cause false positive results or that PC are missed. Moreover, mpMRI has the disadvantage of additional costs (e.g. Gadolinium contrast has to be purchased) and the need for more experienced readers (it may be difficult to draw the right conclusion from the interpretation of all the different sequences that sometimes show conflicting results). Another disadvantage of mpMRI is the long scanning time of about 45 minutes. This becomes a problem as urologists are increasingly convinced about the usefulness of prostate MRI and the number of requests for imaging is growing. To further increase the adoption of prostate MRI by urologists and radiologists while avoiding long waiting lists, it was necessary to modify mpMRI into a scan protocol that was quick and simple.

Therefore, since 2015 attempts have been made to reduce the number of scan sequences. The ESUR and American College of Radiology (ACR) published a second version of the prostate MRI guidelines [9] and in these new guidelines, the role of DCE in the interpretation of prostate MRI is reduced (although it is still recommended to be scanned as part of a mpMRI) and MRSI is no longer recommended. Omitting MRSI and DCE considerably shortens the prostate MRI examination time and reduces costs [10].

In recent years the quality of the DWI sequences on the newest MRI equipment have improved impressively and recent literature data show that DCE has currently limited added value over T2-WI and DWI [11,12,13,14,15]. Even an ultrashort scan protocol limited to only transverse T2-WI and DWI showed similar diagnostic performance as mpMRI for the detection of clinically significant PC, but with the advantage of reducing scan time with about 15 minutes (from about 25 minutes to 10 minutes) [16].

Currently, a scan protocol consisting of only T2-WI (preferably multiplanar) and DWI seems to be the best combination as a short *standard protocol* in daily routine for performing prostate MRI within a short time frame (about 20 minutes) and offering high sensitivity for detection of aggressive PC in patients with elevated PSA. The addition of DCE and/or MRSI may be preserved for other clinical indications, such as detection of PC recurrence after treatment (radiation therapy, radical prostatectomy, brachytherapy, HIFU …) or in doubtful or in complicated cases (e.g. patients with a previous indeterminate MRI, or patients with continuously rising PSA despite negative MRI findings). The decision which scan sequences are needed should be made by the radiologist and may be determined before the examination based on the clinical question and the information provided by the referring clinician, or may be delayed to after completion of the T2-WI and DWI, based on their image quality and diagnostic interpretation.

In the radiological community the debate is still ongoing about the need of using an endorectal coil and/or the required magnetic field strength that is necessary for performing high quality prostate MRI. It is generally accepted that both 1.5 Tesla and 3.0 Tesla scanners can provide adequate and reliable diagnostic quality when the acquisition parameters are optimized and appropriate technology is employed [9].

The use of an endorectal coil likely also improves the diagnostic performance at 3.0 Tesla [17,18] but Barth et al. showed that the image quality of T2-WI was comparable to 3.0 Tesla without endorectal coil and that the endorectal coil insertion caused low to moderate discomfort and pain to the patients, which were arguments for omission of the endorectal coil at 3.0 Tesla [19]. Anyhow, Bratan et al. [20] showed that PC detection rates are not significantly influenced by field strength nor coils used for imaging, but more importantly by PC characteristics such as tumour volume, Gleason score, architecture and location.

## Diagnostic Accuracy of Prostate MRI in the Diagnosis of PC

The determination of the diagnostic accuracy of prostate MRI is dependent on the histopathological gold standard that is used as a reference and on the threshold for a *positive* MRI that is applied when interpreting the images.

The histopathological gold standard has changed over the years. In the past, urologists aimed at detecting and treating any PC of any size and any Gleason score, but since the turn of the century, it became obvious that this resulted in over-diagnosis and overtreatment of indolent PC. As a consequence, the concept of *clinically significant PC* was introduced, referring to the proportion of PC that are deemed aggressive and need treatment, and the notion of *insignificant PC* referring to the proportion of PC that are unlikely to progress to biologic significance and in which *active surveillance* may be offered as treatment option. There is, however, currently still lack of consensus among urologists and pathologists about what exactly constitutes a *clinically insignificant* PC and therefore the *true* diagnostic accuracy of prostate MRI remains difficult to determine [21].

The threshold for scoring prostate MRI *positive* or *negative* may also influence the accuracy in the detection of clinically significant PC. The reported prostate MRI conclusion is not always straightforward (*completely normal* versus *very suspicious*) because doubtful findings or conflicting results between the different imaging modalities may occur. The 5-point Prostate Imaging Reporting and Data System (PI-RADS) scoring system, introduced in the prostate MRI guidelines of 2011 [3,9,22] entails assignment of separate scores for each of the scanned MRI sequences and provides explicit verbal descriptions on how to generate them. Each exam is assigned with an overall assessment score ranging from 1 (indicating that clinically significant PC is highly unlikely to be present) to 5 (indicating that clinically significant PC is highly likely to be present). An overall assessment score of 3 indicates equivocal findings and this enables the radiologist to communicate doubtful findings to the referring clinician. The application of an equivocal score 3 increases the specificity of findings classified as 1, 2, 4 or 5 but it may be a source of confusion to clinicians [21,23] because this indeterminate prostate MRI result often needs to be translated into a binary clinical decision, i.e. to biopsy or not, in patients with elevated PSA. The recommendation to biopsy then depends on whether an overall assessment score of 3 or 4 is used as threshold for a *positive* MRI [24,25].

The PC volume and Gleason score highly influence the detectability on prostate MRI with larger tumours being detected more easily than smaller ones [20,26,27]. Very small tumours <1 mm diameter are below the detection limit of MRI but a PC of 1 cm diameter (0.5 ml) is well within the spatial resolution of T2-WI [20,26,27,28,29]. Higher grade PC are histologically associated with more pronounced destruction of the normal glands, more solid areas of tumour cells and less fluid content as compared to lower grade PC, and consequently show a higher detectability on MRI [20,26,27,28].

The true strength of prostate MRI lies not in the detection of any PC including very small or low-grade lesions, but in the detection or exclusion of the aggressive tumours.

The reported detection rates of prostate MRI range from 100% for Gleason ≥8 PC with size >2.0 ml to 21–29% for Gleason 6 PC measuring <0.5 ml [20,26,27]. The reported sensitivities and specificities of prostate MRI are 80–90% and 47–61% respectively, for detection of high-grade PC and 71–84% and 33–70%, respectively, for detection of PC of any grade [11,15,30,31,32,33,34,35,36,37,38,39,40,41,42]. Negative predictive values of 92–100% are reported for clinically significant PC and 63–91% for PC of any grade [11,30,31,32,33,34,35,36,43,44,45,46,47,48]. An analysis of PC that were missed on mpMRI [49] showed that 67.7% of the missed PC were low grade and 96.6% were organ-confined. With a multiparametric scan protocol, the detection rates for clinically significant PC in biopsy-naive males and men with prior negative biopsies, widely range from 44% to 87% and negative predictive values ranging from 63% to 98%, with trends depending mainly on the definition of clinically significant PC that was applied [46]. A short prostate MRI scan protocol consisting of only T2-WI and DWI seems not to show inferior diagnostic accuracy as compared to mpMRI [16].

In summary, although there is a high variability between the reported accuracies due to differences in definition of the histopathological reference and threshold for a positive MRI that are used, in general the detection rate of prostate MRI for *any PC* is rather moderate, whereas the detection and exclusion of high grade and large volume PC is very high to excellent.

## Clinical Role of MRI in the Detection of PC

**Figure 1:**
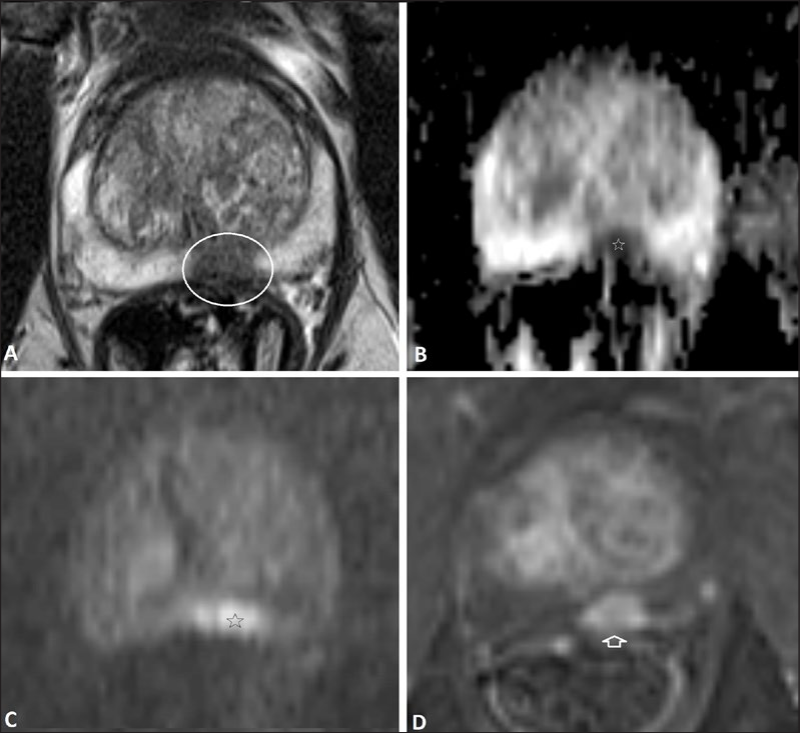
60-year-old men with elevated PSA (20 ng/ml). On axial T2-weighted image **(A)** A nodular lesion with low SI is noted (white circle) posteriorly in de peripheral zone of the mid prostate. This nodule shows low ADC value **(B)** and High SI on high-b-value image **(C)** of DWI, and strong Gadolinium contrast enhancement **(D)**, suspicious for a high-grade prostate cancer, PI-RADS overall assessment score 5. A targeted transrectal ultrasound guided biopsy was performed, confirming a Gleason 4 + 4 prostate cancer.

**Figure 2:**
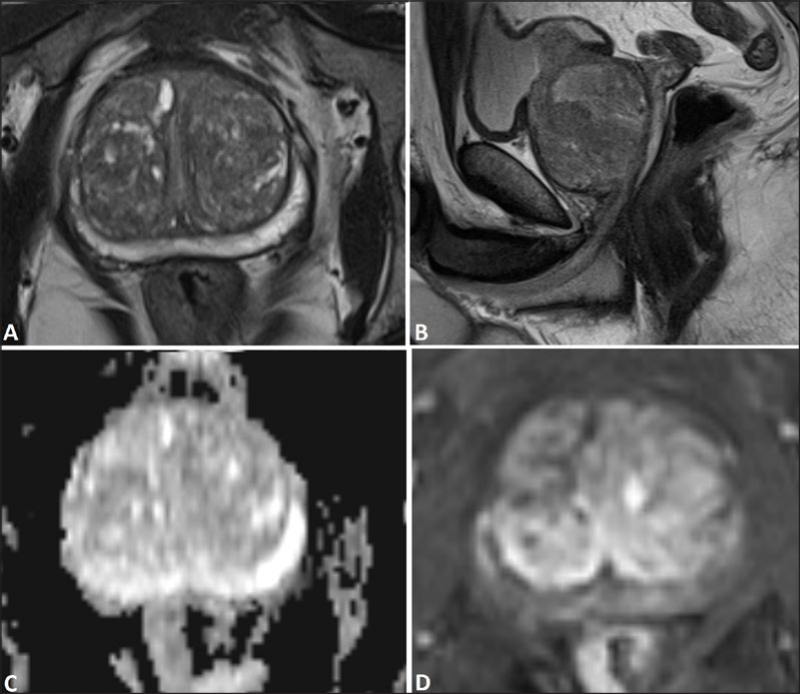
52-year-old man with elevated PSA (12.8 ng/ml). On T2-weighted images (**A:** axial; **B:** sagittal) the prostate is enlarged with estimated volume of 151 ml. The peripheral zone shows a normal high signal intensity. On the ADC map of the DWI **(C)** There are no lesions with restricted diffusion and on the subtraction image of the dynamic contrast enhanced series **(D)** No suspicious contrast enhancing lesions are demonstrated. The PI-RADS overall assessment score is 1 and no biopsy was performed. The patient was followed clinically and with repetitive PSA samplings for more than 4 years and no clinically significant prostate cancer appeared. The elevated PSA level was caused by benign prostatic hyperplasia.

The clinical role of prostate MRI has evolved in the last decade. In the past, prostate MRI was mainly used to visualize biopsy-proven PC for staging purposes to inform the surgeon before radical prostatectomy, about the extent of the disease or to help the radiation oncologist to delineate the clinical target volume [1,2]. The ability of prostate MRI to detect high grade and large volume PC may help to discriminate clinically significant PC from indolent or absent cancer before the biopsy which has led to a refined diagnostic pathway [50]. Prostate MRI may improve the diagnosis of clinically significant PC by serving as an additional decision tool to triage patients with elevated PSA towards immediate biopsy or not. When a suspicious lesion is demonstrated on pre-biopsy prostate MRI, the likelihood of a high-grade PC is high and consequently a targeted biopsy to the suspicious lesion may be performed (Figure 1). When the imaging findings are normal on pre-biopsy prostate MRI, the risk of a high-grade PC is very low (Figure 2). Since the growth and stage progression of PC tend to be slow, consideration could reasonably be given to deferring or even omitting the biopsy in these patients although continued monitoring with repetitive PSA sampling, DRE and/or MRI remains necessary [25,43,44,51,52,53,54,55].

Currently the implementation of prostate MRI in the international guidelines is limited.

The EAU (European Association of Urology) and NCCN (National Comprehensive Cancer Network) guidelines recommend prostate mpMRI only before repeat biopsy, when clinical suspicion of PC persists in spite of initial negative biopsies [3,43,56,57,58,59]. However, the arguments for performing prostate MRI before initial biopsy become stronger. It has been shown that the implementation of pre-biopsy MRI (with consequently targeted MR-guided biopsy in case of a positive MRI or no biopsy in case of a negative MRI) in men with elevated PSA results in an equal to slightly higher detection rate of clinically significant PC, 25–30% reduction in the number of men biopsied, and 10% fewer men being attributed the diagnosis of clinically insignificant PC [24,60,61,62]. Two randomized controlled trials on the use of mpMRI in biopsy naive patients showed conflicting results [63,64] but multicentre controlled studies are pending, such as the PRECISION trial (international study in which Ghent University Hospital participates) and the MRI-FIRST trial. These studies are mainly performed in academic centres with radiological expertise but a problem is that worse results are observed in centres with limited experience [65]. Tonttila et al. [61] performed a prospective randomized controlled trial concluding that mpMRI before biopsy did not improve PC detection rates but the mpMRI were not scanned or reported according to the PI-RADS standards and the urologist who performed the targeted biopsies was unexperienced and used a cognitive approach. Branger et al. [48] reported that a negative MRI is no warrantor for absence of significant cancer but most of the MRIs were done externally in differing hospitals or private practices with limited experience. This variation in image quality and radiologist’s performance in examination interpretation is an important barrier to the implementation of prostate MRI in the guidelines [65].

## Conclusions

The prostate MRI scan protocol has been changing over the years. With the currently available techniques, a short scan protocol consisting of only T2-WI and DWI seems to offer sufficient information and accuracy for most diagnostic indications. Only in doubtful or complicated cases, DCE or MRSI may be performed additionally.

The accuracy of prostate MRI to detect clinically significant PC varies with the applied definition of clinically significant disease. The major value of prostate MRI is to selectively demonstrate or exclude high grade and large tumours. Prostate MRI offers additional information, next to clinical biomarkers such as PSA level and digital rectal examination, to determine the likelihood of a clinically significant PC. Prostate MRI may improve the diagnosis of clinically significant PC by serving as an additional decision tool to triage patients with elevated PSA towards immediate biopsy or not.
